# Extraction Systems and Analytical Techniques for Food Phenolic Compounds: A Review

**DOI:** 10.3390/foods11223671

**Published:** 2022-11-16

**Authors:** Antonio Lama-Muñoz, María del Mar Contreras

**Affiliations:** 1 Departamento de Cristalografía, Mineralogía y Química Agrícola, Universidad de Sevilla, C/Profesor García González, 1, 41012 Sevilla, Spain; 2 Department of Chemical, Environmental and Materials Engineering, Centre for Advanced Studies in Earth Sciences, Energy and Environment (CEACTEMA), Universidad de Jaén, Campus Las Lagunillas, s/n, 23071 Jaén, Spain

**Keywords:** analysis, extraction, green technologies, mass spectrometry, phenolic compounds

## Abstract

Phenolic compounds are highly valuable food components due to their potential utilisation as natural bioactive and antioxidant molecules for the food, cosmetic, chemical, and pharmaceutical industries. For this purpose, the development and optimisation of efficient extraction methods is crucial to obtain phenolic-rich extracts and, for some applications, free of interfering compounds. It should be accompanied with robust analytical tools that enable the standardisation of phenolic-rich extracts for industrial applications. New methodologies based on both novel extraction and/or analysis are also implemented to characterise and elucidate novel chemical structures and to face safety, pharmacology, and toxicity issues related to phenolic compounds at the molecular level. Moreover, in combination with multivariate analysis, the extraction and analysis of phenolic compounds offer tools for plant chemotyping, food traceability and marker selection in omics studies. Therefore, this study reviews extraction techniques applied to recover phenolic compounds from foods and agri-food by-products, including liquid–liquid extraction, solid–liquid extraction assisted by intensification technologies, solid-phase extraction, and combined methods. It also provides an overview of the characterisation techniques, including UV–Vis, infra-red, nuclear magnetic resonance, mass spectrometry and others used in minor applications such as Raman spectroscopy and ion mobility spectrometry, coupled or not to chromatography. Overall, a wide range of methodologies are now available, which can be applied individually and combined to provide complementary results in the roadmap around the study of phenolic compounds.

## 1. Introduction

The term “phenolic compounds” refers to a heterogeneous family of secondary phytochemicals and includes all those substances that have several phenol functions bonded to aliphatic or aromatic rings [1]. Only some phenolic compounds of the phenolic acid family are not polyphenols, but monophenols. Phenolic compounds occur in the plant kingdom, considered secondary metabolites of plants that pass to the animal world by their ingestion [2]. Phenolic compounds are produced de novo by plants and are genetically regulated, both qualitatively and quantitatively, although environmental factors also exist at this level. In addition, they act as protectors against UV radiation and as phytoalexins (injured plants secrete this type of compounds to defend themselves against microbial invasion, such as fungal or bacterial attacks) [3] and contribute to the pigmentation of many parts of the plant (e.g., anthocyanins are responsible for the colour blue, red, orange, purple, etc. that we find in the outer parts of fruits and vegetables). On the other hand, when phenolic compounds are oxidised, they are converted into quinones, which are related to the brown colour that is often undesirable.

Phenolic compounds are found in almost all foods of plant origin and in the by-products resulting from their processing. Foods rich in phenolic compounds are onion, tea, red wine, cocoa, virgin olive oil, etc. [4,5]. These compounds have an impact in the quality, acceptability, and stability of foods, since they act as colorants, antioxidants and provide flavour. Thus, for example, olives contain phenolic compounds that pass into the oil in a small proportion during the extraction period. Virgin olive oil is almost the only oil that contains significant amounts of natural phenolic substances, since the rest of the edible oils, when consumed refined, lose these compounds. For this reason, virgin olive oil has a characteristic flavour, where some phenolic compounds contribute to, that is imperceptible in refined oil.

Phenolic compounds constitute a very complex and structurally diverse fraction made up of many compounds, some of which have not yet been identified. According to their chemical structure there exists two large groups: simple phenolic compounds (non-carboxylic phenols with C6, C6-C1 and C6-C2 carbon skeletons, and carboxylic phenols, e.g. C6-C1 benzoic acid derivatives, C6-C2 phenylacetic acid and derivatives, and C6-C3 cinnamic acid derivatives) and flavonoids (C6-C3-C6) (anthocyanins, flavones, flavanones, flavanols, condensed tannins, among others), along with lignans, stilbenoids (C6-C2-C6), etc. [6]. Due to their important applications in the prevention of diseases because of their beneficial properties, mainly antioxidant activities, studies in relation to their extraction and analysis methods, both qualitative and quantitative, arouse great interest among researchers and numerous papers have addressed this issue [7,8]. Therefore, the aim of this review is to collect some of the most recent literature on the characterisation and analysis techniques, as well as on the extraction methods focusing on the more novel and emerging technologies used for the recovery of these high added-value compounds. As a novelty, this study reports new information about both extraction and characterisation technologies, overviewing a wide range of possibilities for the application in the analysis of phenolic compounds in foods, nutraceuticals and medicinal plants.

## 2. Techniques and Extraction Systems for Phenolic Compounds

Natural phenolic compounds represent a very complex family of compounds that range from monomers such as phenolic acids to highly polymerised molecules, e.g., tannins. To this complexity is added the fact that in nature they are mainly found in conjugated forms with one or more sugar units (monosaccharides, disaccharides and even oligosaccharides) bonded to hydroxyl groups. It is also commonly associated with other compounds such as carboxylic acids, amines, and lipids (e.g., terpenes) as well as linkages with other phenols. Consequently, and considering the complexity of plant matrices too, the extraction methods have received special attention in recent years because of the interesting bioactive properties, health benefits and potential applications of phenolic compounds [9]. Extraction is the main stage for the recovery and purification of phenolic compounds from plant materials before analysis [10] and application because it enables to concentrate these compounds and reduce interfering components. The extraction process of phenolic compounds is especially critical when performing quantitative analyses since their tissue distribution in plants is not uniform. At the cellular level, the hydrophilic phenolic compounds (e.g., phenolic acids, anthocyanins, and low molecular weight tannins) are mainly located in the vacuoles, while insoluble phenolic compounds (e.g., condensed tannins, phenolics covalently bound to insoluble polymers such as polysaccharides or proteins forming stabilised macrocomplexes) are usually found in the cell walls. The latter type is usually known as bound phenolic compounds, and they have been the objective of numerous studies in recent years as reviewed by Rocchetti et al. [11]. In fat systems such as oils, it also becomes complex as bound phenolic compounds can also be associated with glycerides by intermolecular forces, including hydrogen bonds and hydrophobic interactions [12].

Traditionally, the extraction of phenolic compounds, especially free phenolic compounds, has been carried out using different conventional solid–liquid extraction techniques (e.g., Soxhlet, maceration, and hydrodistillation) in which water and organic solvents, such as acetone, ethanol, methanol, and ethyl acetate, among others, are used. The quantitative and qualitative performance of the extraction depends to a great extent on the polarity of the solvent used and there is no defined method and solvent. The extraction performance will depend on the chemical composition of the phenolic compounds to be extracted and the number and position of their hydroxyl groups, molecular size, as well as other factors such as temperature, contact time, particle size, and interaction with other food components, among others. Although these conventional or traditional extraction techniques are simple procedures, they have a series of drawbacks: low selectivity and low recovery percentages or extraction yields; they are very laborious and time-consuming; they also use large amounts of organic solvents that in many cases can be toxic and may remain in trace quantities in the extracts.

Due to these drawbacks, in recent years these conventional techniques are being replaced by alternative extraction methods, which generally use an energy source to increase the transfer of the phenolic compounds to the solvent. In addition, techniques that are eco-friendly and require lesser amounts of solvents have also been sought. It is in this sense that, in last decade, new systems and emerging techniques have been developed and implemented to reduce the amount of sample to be treated, the use of harmful and pollutant solvents, the extraction time and the energy consumption. Recent advances in this field include improvements and variants of traditional solid–liquid, liquid–liquid and solid-phase extraction techniques [13] and among the non-conventional or modern extraction techniques, the use of ultrasound, microwaves, supercritical fluids, and pressurised fluids should be highlighted. These novel extraction methods are used for extracting phenolic compounds from a wide variety of different plant species, such as garlic, onion, leek, oregano, peppermint, rosemary, salvia, olive leaves, etc. [14]. Table 1 shows the advantages and disadvantages of the main extraction technologies used for obtaining phenolic compounds from plant materials with a brief application description and Table 2 shows some examples of their use in specific matrices.

### 2.1. Liquid–Liquid Extraction (LLE)

The extraction of phenolic compounds from plant materials usually requires a solid–liquid extraction as a first step followed by a direct liquid–liquid extraction of the clarified primary aqueous extract using organic solvents including petroleum ether and ethyl acetate to fractionate the phenolic compound fraction [29]. Phenolic compounds can either be separated either by a continuous liquid–liquid extractor that enables self-sustained extraction of the aqueous extract with an immiscible solvent in the presence of a heat source [30] or by a separating funnel in which the extract is vigorously shaken together with an immiscible organic solvent. The solubility of the phenolic compounds in the solvent plays a decisive role in this type of extraction. This solubility is conditioned by the chemical nature of the phenolic compounds, and can vary according to the family to which they belong. For example, the extraction of polar phenolic compounds such as phenolic acids need a mixture of organic solvents with different proportions of water to increase the polarity of the solution. Other factors such as miscibility, density, pH, temperature, extraction time, number of successive extractions, and the extract-to-solvent volume ratio also determine the recovery of phenolic compounds [31].

Although LLE using organic-aqueous liquid–liquid biphasic systems is still one of the most common solvent extraction techniques used to recover phenolic compounds from plant materials. However, according to the disadvantages already mentioned with regard to the use of volatile and organic solvents, these systems are being substituted by aqueous two-phase extractions (ATPE) [32] to reduce the toxicity. ATPE are liquid–liquid biphasic systems formed by the combination of hydrophilic substances, such as polymers [33] and alcohols [34], at precise concentrations with a salt which results in the formation of two hydrophilic phases very useful for the separation of phenolic compound from vegetable tissues (carrot, wood, fig fruits, etc.). On the other hand, LLE is a time-consuming technique. To overcome this disadvantage, recent research has focused on the development of microextraction techniques such as dispersive liquid–liquid microextraction (DLLME) and sugaring-out liquid–liquid extraction. Specifically, DLLME is performed by injecting a mixture of an appropriate extraction solvent (e.g., ethyl acetate) and a dispersive solvent (acetone, acetonitrile) into the aqueous plant extract. The resulting solution is then centrifuged to separate the organic and aqueous layers. This novel technique has been optimised to extract phenolic compounds from different cultivars of plum leaves [35]. The liquid biphasic flotation (LBF) system is another emerging and modern liquid–liquid extraction method that faces the difficulties of LLE regarding separation efficiency and low extraction yields. Chia et al. [36] have applied this novel extraction method with low-cost equipment to extract phenolic compounds from kesum plant. The liquid biphasic system integrates the techniques of ATPE [37] and solvent sublation (a type of adsorption bubble separation technique) [38].

### 2.2. Ultrasound-Assisted Extraction (UAE)

The physical phenomena that affect the extraction of phenolic compounds are influenced by sonication since these are normally found inside the cells of the plant tissues. In ultrasound-assisted extraction, high-frequency sounds (between 20 kHz and 40 kHz) are used to release phenolic compounds from the plant material [39,40]. As a result of the rapid formation and collapse of bubbles in the solvent caused by waves of high-frequency sound and the pitting of the plant matrix surface because of the forces of repeated cavitation in the surrounding solvent, phenolic compounds quickly diffuse throughout cell walls towards the solvent. Thos improves extraction efficiency compared to conventional extraction procedures [41], without significantly altering the properties of the phenolic extracts, allowing the separation of phenolic compounds of pharmacological and industrial interest from plant by-products, such as olive pomace [42]. However, in the case of antioxidant activity, a property of great importance in the case of phenolic compounds, it must be considered that the influence of the application of ultrasound waves on the extraction medium and the matrix may favour not only the extraction of phenolic compounds, but also the development of secondary reactions that generate a decrease in the phenolic concentration, an undesired effect at the industrial level. By reducing the particle size of the plant material, the area of exposure to the solvent and the cavitation produced is increased. Nonetheless, it is generally used at lower temperature than other technologies, reducing the thermal damage of most thermolabile compounds and making it a cost-effective technique [43]. Ultrasounds also facilitate tissue rehydration if dry materials are being used to open the pores, which in turn increases the mass transport of soluble constituents by diffusion and osmotic processes. Another feature according to Irakli et al. [44], is that UAE is one of the cheapest techniques and has the lowest instrumental requirements among the non-conventional extraction methods developed, it is easy to scale up and well-established at the industrial level.

The most influential parameters in the application of ultrasound-assisted extraction are time, temperature, amplitude, and sonication power [45,46,47]. The influence of the extraction solvents has also been assessed [48]. Under optimal conditions of the former parameters, UAE has been shown to be more efficient in the recovery of total phenolics, flavonoids, proanthocyanidins and antioxidants in tea tree leaves [41] in comparison with conventional extraction techniques. The results reported by Ferarsa et al. [49] indicated that a suitable ultrasonic pre-treatment can enhance the maceration process for the recovery of anthocyanins and other phenolic compounds from the peel and pulp of purple eggplant, reducing the extraction time and increasing the yield of phenolic compounds.

### 2.3. Microwave-Assisted Extraction (MAE)

MAE has also been developed as another novel technique for obtaining phenolic compounds from a considerable number of plant matrices (blueberry leaves, fenugreek seeds, hibiscus flower, rice grains, orange peels, brown macroalgae, etc.) [50,51]. The sample is extracted by applying microwave energy in a suitable solvent. This technique depends on the matrix and limits the solvents that can be used, since they should not be transparent to microwave radiation and must have a high dipole moment, without forgetting the solubility of the phenolic compounds in them. Microwaves are high-frequency radiations (0.3–300 GHz) in the form of electromagnetic waves whose wavelength range from 1 mm to 30 cm. Unlike other extraction processes, where heat is transferred from the outside to the inside of the plant matrix, microwave energy heats the sample in a simultaneous and homogeneous way. Microwave energy causes the heating of intracellular water and the rupture of the plant cells, thus facilitating the transfer of phenolic compounds to the solvent and its penetration into the plant matrix. This type of radiation allows for faster and more efficient extraction and selective heating of the sample according to the solvent used. Therefore, microwave energy accelerates the extraction and improves the yield using a smaller volume of solvent. Extraction yield is determined by the type of solvent, temperature, microwave power, irradiation time and characteristics of the plant material, mainly particle size and distribution. Undoubtedly the main advantage reported for MAE is the significant reduction in extraction time compared to conventional heating [52]. For example, Vieira et al. [53] compared maceration and MAE to maximise the extraction of phenolic compounds from walnut leaves and under optimum conditions they observed that MAE required only 3 min against almost 2 h for maceration.

### 2.4. Supercritical Fluid Extraction (SFE)

This technique is based on the fact that the solubility of a substance strongly depends on the solvent density [54]. Thus, one of the main features of a supercritical fluid is the possibility of modifying its density by adjusting the temperature and/or pressure. A supercritical fluid occurs when a fluid is applied under pressure and temperature at values higher than its critical point, without phase transition occurring, in which the properties of the fluid become indistinguishable from the liquid and vapor phases. Supercritical fluids have densities similar to liquids but with higher diffusion coefficients and lower viscosities, similar to gases, making extraction faster and more selective than with a liquid organic solvent. SFE is a suitable extraction technique for the recovery of non-polar phenolic compounds. Due to its low polarity and high diffusivity, carbon dioxide has been the fluid of choice in most extractions as it is a suitable solvent for nonpolar analytes. Thus, for more polar phenolic compounds it is necessary to introduce co-solvents and/or modifiers such as ethanol, methanol, or water to increase the polarity of CO_2_ and improve the extraction of these compounds [55]. Pinto et al. [56] optimised the extraction of phenolic acids and tannins from the underexploited by-product of *Castanea sativa* shells using SFE-CO_2_ with ethanol as a co-solvent. The authors observed a significant increase in the extraction yield and DPPH% scavenging activity when 15% ethanol was added.

SFE is considered an eco-friendly and sustainable extraction methodology for obtaining phenolic extracts from plant materials, with hundreds of species reported in the literature [57], since the extracts are free of residual solvents. The use of CO_2_ can be recycled thus making it another sustainable strategy. SFE has been successfully used for the extraction of phenolic compounds, anthocyanins, flavonoids from purple corncob [58], peach leaves [59] and antioxidant phenolic compound-rich extracts from Andes berry residues and rosemary, which can be used for food, feed, and medicinal applications [60].

### 2.5. Pressurised Liquid Extraction (PLE)

This technique is also known as accelerated solvent extraction (ASE), although many other terminologies are also used, for example, when the solvent is water other common terms, such as subcritical water extraction (SWE) or pressurised hot water extraction (PHWE) can be found in the literature [61]. It deals with a solid–liquid extraction method which is performed in an automated system at elevated temperatures (maximum of 200 °C) and pressures (around 1700 psi) to achieve extractions in very short periods of time. For example, Santos et al. [62] extracted the highest content of anthocyanins and other phenolic compounds from 5 g of jaboticaba skins in less than 10 min, consuming a very small volume of solvent. Samples are extracted in the absence of oxygen and protected from the light. High temperatures and pressures facilitate the extraction of the trapped phenolic compounds, forcing the solvent to penetrate the pores of the matrix more easily and increasing its capacity to solubilise them allowing for faster diffusion rates and accelerating the kinetics of the extractive process [63]. High temperatures reduce solvent viscosity and break solute–matrix interactions (dipole–dipole attractions, hydrogen bonds and van der Waals forces). Operating at elevated pressures prevents the solvent from reaching its boiling point, which provides faster extractions of the phenolic compounds. For beetroot waste, a pressure increase from 7.5 to 10 MPa significantly increased the extraction yield when using ethanol as the solvent [64]. This system has been shown to be very useful for the extraction of phenolic compounds, flavonoids and catechins from olive leaves [65], avocado peel by-product [66], and waxy barley [67], among many other plant materials. However, one of its disadvantages in food applications is that undesirable compounds, such as 5-hydroxymethylfufural, can be generated at greater levels than, for example, other thermal technologies, such as MAE [52].

### 2.6. Novel Extraction Solvents for Extracting Phenolic Compounds

Organic solvents and their combination with water are common solvents used to extract phenolic compounds from plant materials. They play a fundamental role in the efficiency of extraction [68] and are usually consumed in large quantities in the separation processes. As previously mentioned, most of them are volatile organic compounds and come from unrenewable resources. This is not recommended from the point of view of current sustainable development goals and green chemistry because, in many cases, they are toxic and highly polluting due to their high volatility, although they can be recycled by distillation. For this reason, scientists are exploring novel and greener solvents with better environmental, health and safety profiles. Besides CO_2_ and water, whose extraction properties can be modified by temperature and pressure, novel solvents are being investigated. An example of compounds that meet these characteristics are deep eutectic solvents (DES) and ionic liquids (IL), two main classes of innovative solvents that have recently emerged as a competitive alternative to replace the conventional toxic organic solvents in the extraction of phenolic compounds from plant materials, agro-food industry by-products and industrial wastewaters (apricot pulp, onion, olive, tomato, pear, etc.) [69,70,71] due to their availability, very low volatility, biodegradability, low toxicity, safety, reusability, and low-cost. DES are a new generation of fluids formed from a mixture of a hydrogen bond acceptor (e.g., choline chloride) and a hydrogen bond donor compound (e.g., glycerol) in a solid state that associate by hydrogen bonding. DES have a lower melting point than that of each individual component and are liquid even at very low temperatures. H-bonding interactions are responsible for the high extractability of DES [72]. Of special interest are natural DES or NADES, which can even be present in food products without further purification of the extracts if they are, for example, safe for consumption and their use is approved by food safety administrations [73]. The properties of the DES solution can be modulated by changing its composition (compounds mixed and ratio) to increase the efficiency of the extraction of phenolic compounds, both free and bound ones [74].

IL are designer non-flammable solvents that have high thermal and chemical stability and have no detectable vapor pressure. IL are salts that consist of an organic cation (e.g., alkylammonium) and an organic or inorganic anion (e.g., NO^3−^), whose physicochemical properties (density, melting point, viscosity, etc.) can be modulated. DES and IL have been used alongside modern extraction techniques, such as UAE [75] and MAE [76], for improving extraction yield, while shortening the processing time, and increasing the stabilisation capacity of phenolic compounds from plant and food matrices, such as *Moringa oleifera* L. leaves, olive leaves, and rosemary leaves [77].

### 2.7. Solid Phase Extraction (SPE)

In general, during liquid–liquid and solid–liquid extractions of phenolics from plant matrices, other undesirable compounds such as sugars, proteins and organic acids can also be extracted at the same time and lead to possible interactions and the formation of insoluble complexes. Therefore, an additional step for eliminating interferences and purifying the crude plant extracts is required [28]. The SPE method is the preferred sample preparation technique for the separation and clean-up of different types of compounds. An advantage of SPE is related to the in-line combination of SPE extraction with HPLC and hence the crude extraction of plant material can be directly analysed. In SPE the phenolic compounds are adsorbed in a column packed with functionalised material. Several solid phases or sorbents can be used depending on their selectivity and stability; however, Diol, C8 and C18-bonded silica cartridges [78,79] are the most widely used for retaining target phenolic compounds. Two variants of this technique, known as dispersive and magnetic solid-phase extraction (d-SPE and M-SPE, respectively), have been recently evaluated as clean-up steps for the analysis of phenolic compounds in *Myrciaria cauliflora* (or *Plinia cauliflora*) peel [80] and oilseeds [81]. In d-SPE, the sorbent (e.g., diatomaceous earth) is added directly to the extract and not packed into a column or cartridge while the second one uses magnetic nanoparticles of iron oxide or graphene [82].

### 2.8. Combined Use of Different Techniques

The possibility of combining the previously described techniques or with other extraction procedures have been addressed by numerous studies to simultaneously extract and fractionate phenolic compounds. For example, Palma et al. [83] and da Silva et al. [84] successfully combined and developed an in-line/on-line method based on the coupling of PLE and SPE for the separation of phenolic compounds from grapes and apple pomace, respectively. Other works report the application of PLE assisted by ultrasound to improve and intensify the extraction of phenolic compounds from passion fruit bagasse and pomegranate peel, resulting in an increase in the total phenolic compound yields [85,86]. To reduce the non-polar fraction obtained from the plant material these authors reported using a previous SFE treatment. PLE has also been combined with SFE in an integrated downstream process for recovering antioxidant phenolic compounds from *Sida rhombifolia* leaves [87], in which a SFE depressurisation step was studied to improve PLE efficiency and antioxidant potential. These techniques have also been successfully combined by García-Mendoza et al. [88] in a two-stage sequential extraction strategy for producing extracts rich in anthocyanins and heat-resistant phenolic compounds. A new extraction technology based on the combination of SPE/SFE was originally proposed and constructed by Klejdus et al. [89] incorporating a SPE cartridge into a SFE extraction cell for the recovery of different groups of polar phenolic compounds from different plant species, freshwater microalgae and some cyanobacterial species.

## 3. Analytical Tools for the Analysis of Phenolic Compounds

**Figure 1:**
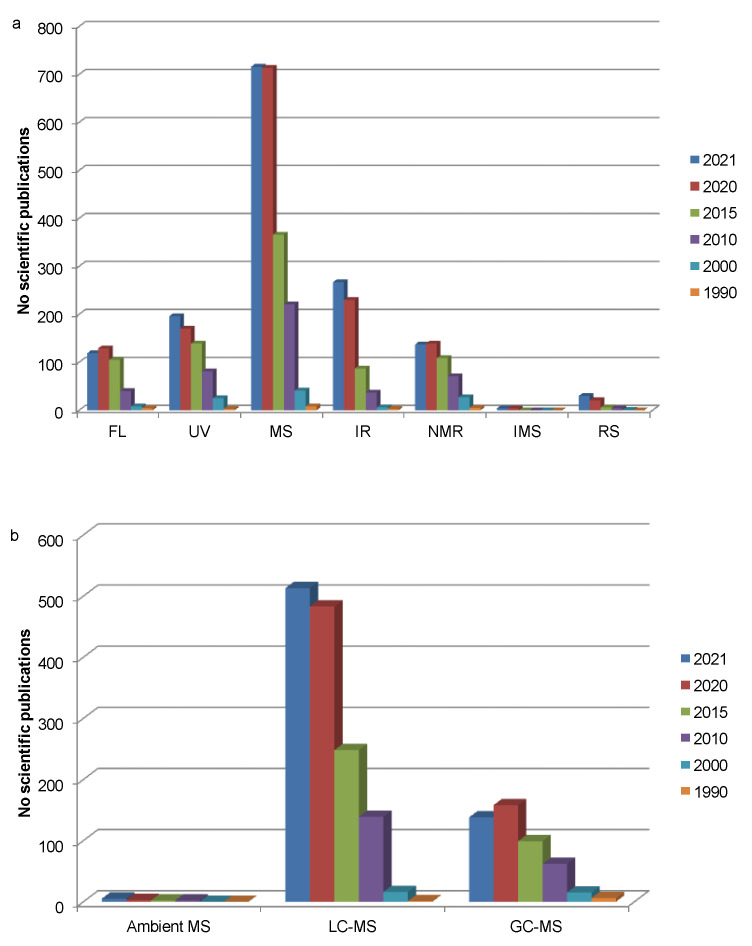
(**a**) Number of publications found in Scopus, which contain spectroscopy tools in the title, abstract and/or keywords of studies on phenolic compounds. The keywords were “phenolic compound” and “fluorescence” (FL), “ultraviolet” (UV), “mass spectrometry” (MS), “infrared” (IR), “nuclear magnetic resonance” (NMR), “ion mobility” (IMS) or “Raman” (RS) were used. (**b**) The keywords were “phenolic compound” “mass spectrometry” and “ambient”, “liquid chromatography” or “gas chromatography”.

There are plenty characterisation technologies which can be applied for the detection and structural elucidation of phenolic compounds, including UV–visible (UV–Vis), fluorescence (FL), infrared (IR), nuclear magnetic resonance (NMR) and Raman spectroscopy, ion mobility spectroscopy (IMS), and mass spectrometry (MS). As a summary, Figure 1 depicts the number of publications on this topic, where it can be seen the evolution since 1990. The application of MS predominates as a characterisation tool of phenolic compounds. They can be applied alone or coupled to a chromatography technique, which in turn enables the separation of the compounds to provide individual information about their spectroscopic properties.

### 3.1. UV–Visible Spectroscopy

**Figure 2:**
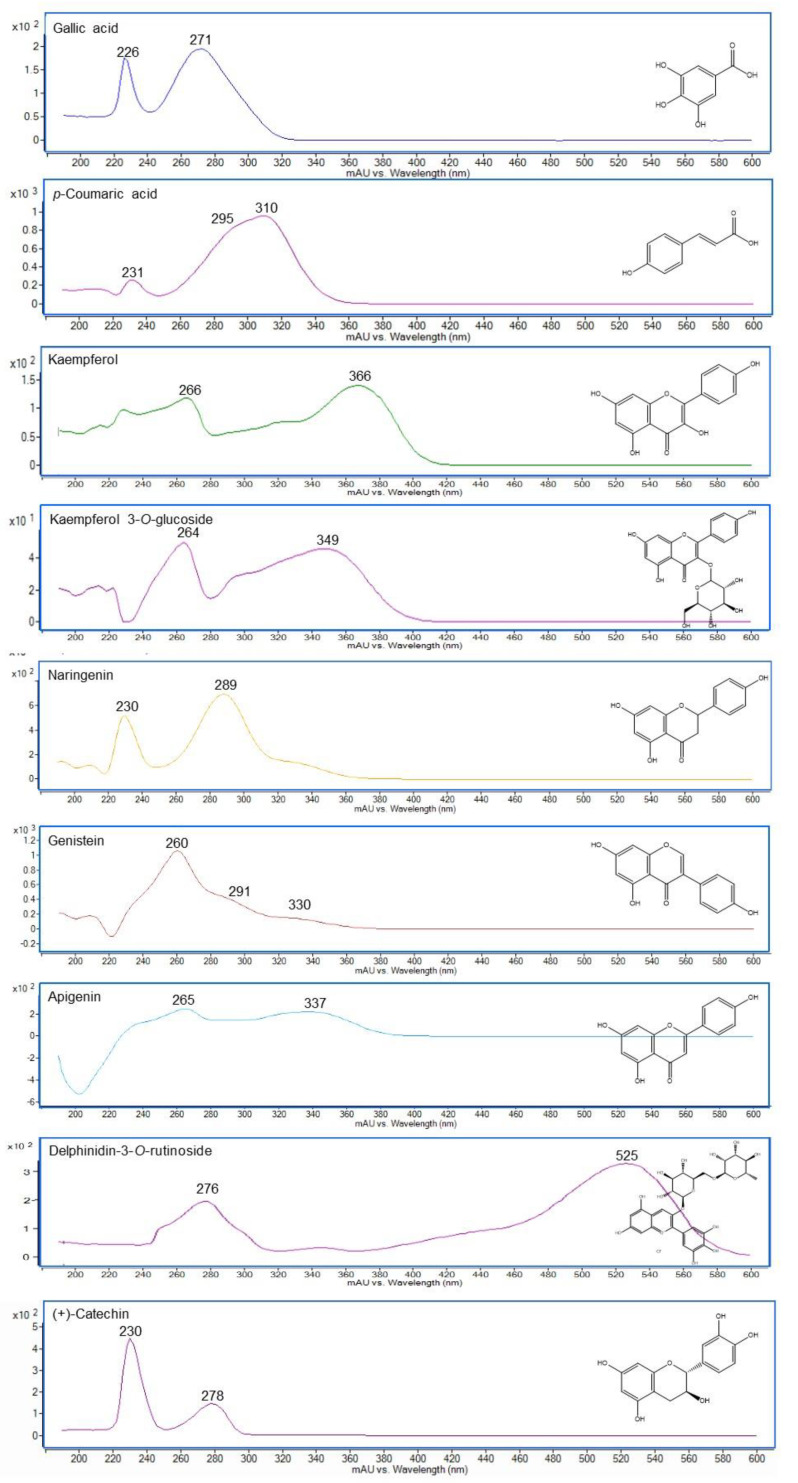
UV–Vis spectra obtained by RP-LC-DAD-QTOF-MS of gallic acid (hydroxybenzoic acid), *p*-coumaric acid (hydroxycinnamic acid), kaempferol and kaempferol 3-glucoside (flavonols), naringenin (flavanone), genistein (isoflavone), apigenin (flavone), delphinidin 3-*O*-rutinoside (anthocyanin), and catechin (flavanol).

UV–Vis spectroscopy can serve as a tool to establish the structural class of phenolic compounds. Spectrophotometric analyses covering the region from 190 to 600 nm is of particular interest to sweep the absorption region of the phenolic compounds. The phenolic moiety confers one or two absorption bands in the UV region. The band 305−390 nm (band I or B) comes from the cinnamoyl part related to the B-ring in the case of flavonoids. The band 230−300 nm (band II or A) correlates with the benzene moiety or A-ring benzoyl counterpart in flavonoids [90,91]. Accordingly, the phenolic class can be classified into two main groups: (1) cinnamic acid derivatives (C6-C3), flavonols and flavones, which exhibit a strong UV band I with a weak band II, and (2) hydroxybenzoic acids (C6-C1), hydroxyphenylacetic acid (C6-C2) and tyrosol (C6-C2), and their derivatives, e.g., hydrolysable tannins [90,91,92,93]. In flavonoids, such as flavanols, flavanones, dihydroflavonols, isoflavones and their derivatives, band II is the main peak in the range from around 260–295 nm, while band I is often found as a little shoulder at 300–330 nm [93,94]. Alternatively, anthocyanins, the main-coloured phenolic compounds, show a strong absorption band at ~530 nm (visible radiation) [95] and thus enabling their selective detection (Figure 2). 

As mentioned before, phenolic compounds can present substitutions in the aromatic ring, and this affects the molar absorptivity and the wavelength at which the maximum occurs for each band. The wavelength depends on the position of the substitution in the aromatic ring, providing a particular value [90] (e.g., see kaempferol and kaempferol 3-*O*-glucoside in Figure 2).

**Figure 3:**
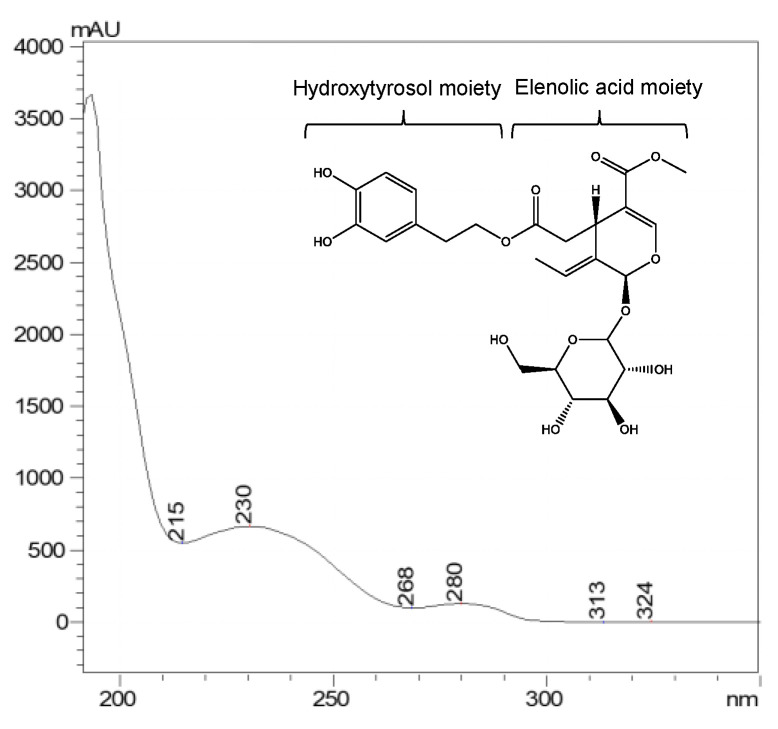
UV–Vis spectra obtained by RP–LC–DAD of secoiridoid oleuropein.

Moreover, the UV spectra of phenolic compounds can also be affected by the presence of certain moieties that provides new absorption bands. For example, the secoiridoid oleuropein absorbs around 240 nm and 280 nm [96,97] due to the presence of an elenolic acid moiety and hydroxytyrosol, respectively [97] (Figure 3). When complex mixtures are studied, their spectra the maximums reflect where the major compounds absorb. For example, in the range between 250–400 nm, an olive leaf extract showed two distinct absorption bands, band II around 280 nm and band I around 325 nm. The former band was mainly due to the presence of hydroxytyrosol and oleuropein and the latter came from flavones and verbascoside [98].

Due to this feature and its reasonable cost, UV detectors and diode array detectors (DAD), which enable the recording of UV–Vis absorption on-line, are widely applied in combination with liquid chromatography (LC) and capillary zone electrophoresis (CZE or CE) for the determination of phenolic compounds. Moreover, for routine analysis and quantification, the wavelength 280 nm can be applied since most phenolic compounds show absorption at this wavelength to a greater or lesser extent [91]. Other usual absorption wavelengths are, e.g., 210 nm for phenolic compounds, 240 nm for secoiridoids, 310–320 nm for hydroxycinnamic acids, 350–370 nm for flavonols, 340–350 nm for flavones, and 520 nm for anthocyanins [91,99,100,101]. Nonetheless, this presents some drawbacks for identification. For example, standards should be analysed to compare both the retention time and UV–Vis spectra with the unknown compound. Furthermore, the electrophoretic/chromatographic step could have insufficient resolving power to adequately separate all phenolic compounds when complex mixtures are analysed, making assignment difficult. This fact explains that the current trend is to apply LC coupled (in-line or on-line) to both DAD and mass spectrometry (MS) to obtain complementary information when phenolic compounds are profiled in complex matrices, such as food plants [100,102,103,104], medicinal plants [105,106] or nutraceuticals [107,108].

### 3.2. Fluorescence Spectroscopy

The majority of phenolic compounds show a FL behaviour since these compounds contain aromatic rings and, in some cases, combined π bonds, absorbing in the 260–310 nm range, which is quite similar to the excitation maximum. The emission occurs in the near-UV range (310–457 nm) [109,110,111]. This phenomenon has been exploited by a recent study applying 280 nm as the excitation wavelength to detect wine phenolic compounds and the emission spectra was recorded between 295–600 nm. It enables the classification of different wines based on their components and applying parallel factor analysis and a soft independent modelling classification analogy as a multivariate analysis. The results showed that the representative loading from λem at 315/317 nm could be related to catechin and epicatechin, 360 nm may result from signals coming from phenolic acids, phenolic aldehydes, and tryptophan, while 327 nm, 303 nm and 411 nm are related to the presence of p-hydroxybenzoic acid, tyrosol and cinnamic acids, respectively [112].

**Figure 4:**
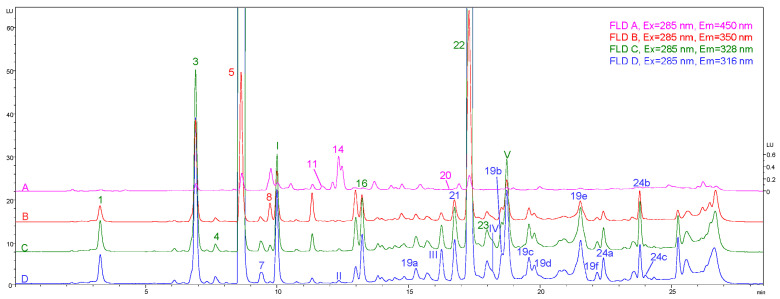
Chromatogram of an olive oil extract (‘Picual’ cultivar) obtained at an λex of 285 nm and different λem wavelengths using RP–LC–FL. Peak identification numbers: (1) oxidised hydroxytyrosol; (2) gallic acid; (3) hydroxytyrosol; (4) 3,4-dihydroxyphenylacetic acid (internal standard); (5) tyrosol; (6) 4-hydroxybenzoic acid; (7) 4-hydroxyphenylacetic acid; (8) vanillic acid; (9) syringic acid; (10) homovanillic acid; (11) *p*-coumaric acid; (12) vanillin; (13) sinapic acid; (14) ferulic acid; (15) *m*-coumaric acid; (16) hydroxytyrosol acetate; (17) oleuropein; (18) *o*-coumaric acid; (19a–f) oleuropein aglycon isomers; (20) luteolin; (21) decarboxymethyl oleuropein aglycon; (22) (+)-pinoresinol; (23) acetoxypinoresinol; and (24) ligstroside aglycon isomers. Adapted from [111].

As before, this technique is coupled to a separation method (e.g., LC and CE) for the characterisation and/or quantification of phenolic compounds, particularly, when analysts look for sensitivity and selectivity, as it is much better than with UV [91,113]. For this purpose, a compromise could be to apply the excitation and emission wavelengths around 280 and 320 nm, respectively [91,114]. Alternatively, a recent study applied “multi-emission” detection by recording four different emission wavelengths simultaneously (316, 328, 350 and 450 nm) [111], increasing the sensitivity and the number of compounds characterised. This enabled the determination of more than 20 phenolic compounds with adequate intensity and selectivity depending on the emission wavelength (Figure 4). Moreover, when the emission of an analyte saturates the detector, another less sensitive wavelength can be selected to widen the dynamic range.

In other studies, normal phase (NP)–LC–FLD has been selected to selectively determine monomeric and oligomeric flavanols using several excitation and emission wavelengths, e.g., λex 230 nm and λem 321 nm for barley and avocado samples [115,116] and λex 280 nm and λem 347 nm for cranberry-based nutraceuticals [113]. This methodology is quite useful in providing the quantity of flavanols oligomers (or procyanidins or condensed tannins) separated as a function of the degree of polymerisation and galloyl units present in their structures [115].

### 3.3. Infrared Spectroscopy

IR region comprises wavelengths between 700 nm and 1 mm and can be divided into near-IR (or NIR) (0.78–2.5 μm or 12,800–4000 cm^−1^), mid-IR (2.5–50 μm or 4000–200 cm^−1^), and far-IR (50–1000 μm or 200–10 cm^−1^) [91]. A compound with the capacity to absorb infrared light will record a characteristic IR spectrum for its discrimination [117]. The advantage of this technology is that it can be applied for fast and non-destructive analyses [115]. Applications include portable NIR to perform on-field prediction of phenolic composition, as some studies have demonstrated in olive fruit and olive oil [118,119].

However, a major disadvantage of this technology is the characteristic overlap and complexity in the NIR spectra that makes interpretation of the data difficult [120]. Mathematical treatment and multivariate analysis can be of help. For example, NIR spectroscopy combined with chemometrics has been applied for samples discrimination. For example, partial least squares (PLS) enabled the grouping of waxy and non-waxy barley extracts through the analysis of flavonoids, including procyanidins, using NIR in the regions of 1415–1512 nm, 1650–1750 nm, and 1955–2035 nm [115].

**Figure 5:**
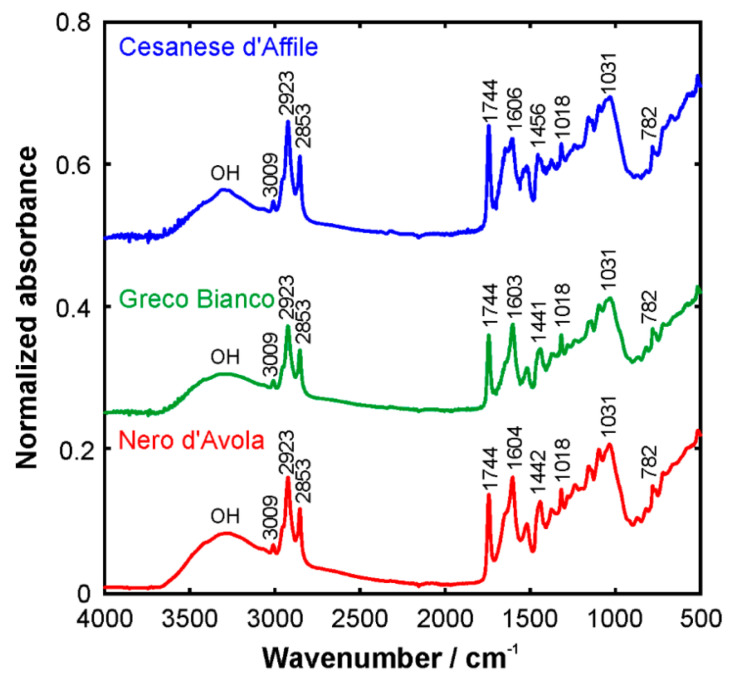
FTIR spectra of three types of grape seeds in the mid-infrared region [122].

Alternatively, for rapid quantification, mid-IR combined again with PLS regression was correlated with oleuropein content in olive leaf determined by HPLC-UV (280 nm) [121]. Additionally, Fourier transform IR spectroscopy in the mid-IR region was applied to profile phenolic compounds along with fatty acids in grape seeds by the analysis of the main functional groups [122]. The bands around 1600 cm^−1^ were related to the stretching of C=OO^−^ and aromatic C=C groups, e.g., in pectins and phenolic compounds, and with the bending vibrations of OH groups. Additionally, these authors suggested that the aromatic C-C stretching at around 1520 cm^−1^ and 1443 cm^−1^, aromatic C-H stretching at 1143 cm^−1^ and rocking of CH_2_ at 782 cm^−1^ could be associated with phenolic compounds (Figure 5). Applications in medicinal plants and nutraceuticals have also been reported in a review by Nagy et al. [123]. In the latter case, NIR is useful not only for the analysis of phenolic compounds, but also for the detection of adulterations and contaminants [123].

Besides the applicability of IR spectroscopy in phenolic compounds analysis, this technique also offers several advantages, including being fast, cost-effective, and non-destructive [91]. However, for a better elucidation of the individual phenolic composition, its coupling with LC requires the elimination of interference from the mobile phase and solvent, which is not an easy task. Recent advances in the interface between LC and IR open new possibilities [117], but studies are still limited in the case of phenolic compounds. For example, a new study applied a self-regulating spray dryer to remove the LC mobile phase and retain the compounds of interests, two furanocoumarin isomers, which were discriminated at the μg level [124].

### 3.4. Mass Spectrometry

Today, MS is an indispensable technique for the structural elucidation and profiling of phenolic compounds. Some of the advantages of this separation and detection technique are its high sensitivity, selectivity, speed, wide dynamic range, etc. [91]. Several mass spectrometers are available, which can be primarily classified as low- and high-resolution MS. Examples of the first type are the quadrupole (Q), ion trap (IT) and triple quadrupole (QQQ), which provide mass errors of around 0.5 Da, while the second includes time-of-flight (TOF), Fourier transform (FT)-ion cyclotron resonance (ICR), and their hybrids such as QTOF and linear IT-Orbitrap, with mass errors lower than 5 ppm. Advantages of the former are the price, being particularly valuable for identification based on databases, e.g., the use of quadrupole is extended when coupled with GC, while IT and QQQ enable fragmentation patterns to be obtained for structural determination. QQQ is also the primary choice for quantification thanks to its selectivity and sensitivity [125]. GC-Q-MS has been applied for the analysis of phenolic compounds, but it generally requires a derivation step [126]. Probably, this fact favours the use of LC with the rest of the mass analysers for the direct analysis of extracts in a liquid state by the means of low severity interfaces. Examples of applications of LC-IT-MS include olive phenolic compounds in food and by-products in untargeted applications [127]. LC–QQQ is commonly applied for the targeted analysis of phenolic compounds, e.g., Gai et al. [128] applied it for the analysis of pigeon pea samples.

Alternatively, high-resolution MS enables the generation of the molecular formula of the compounds with the help of sophisticated software and on the basis of their isotope pattern and accurate *m*/*z* measurements. This provides a high potential for screening purposes. In the case of hybrids with high-resolution characteristics, such as QTOF which enables both the generation of the molecular formula of the compounds and to fragment molecules, its confirmative capacity is the highest [125]. Moreover, the use of QTOF has been extended to elucidate the primary structure of new phenolic compounds, while stereochemistry can be established by NMR [104]. For example, Wang and co-workers have applied a MS-based platform for the profiling of tea seed oils from 15 regions of China, i.e., RP-LC-QTOF-MS was applied to first characterise phenolic compounds and then RP-LC-QQQ-MS was used for quantification purposes. The former enabled the identification of 24 phenolic compounds, including some tea polyphenols, and the latter allowed the distinction of various *Camellia* seed oils [129].

Faster MS methods are being developed to elucidate phenolic compounds in food matrices, including “ambient MS” and direct-infusion MS. The latter has been applied, for example, to identify 11 chemical species, including phenolic compounds, in pineapple at different maturation stages, by using FT-ICR-MS and electrospray ionisation (ESI) in the negative ionisation mode. It enables mass errors lower than 1 ppm to be obtained, giving information about double bond equivalent (DBE) values and molecular formula [130]. “Ambient MS” is based on older ionisation interfaces such as ESI, but the former tends to be performed in an open atmosphere directly on samples with minimal or no sample preparation, or by using auxiliary surfaces [91,131]. This includes ionisation techniques such as direct analysis in real-time (DART)-MS, desorption electrospray ionisation (DESI)-MS, easy ambient sonic-spray ionisation (EASI)-MS and derivative techniques, which has been applied in food quality, authenticity and safety analyses [131,132]. Its application in studies on phenolic compounds is still marginal compared to LC- an GC-coupled MS (Figure 1). In a recent application, DART-Orbitrap-MS was studied to develop a rapid detection (two min) of phenolic compounds for the discrimination of olive oils using principal component analysis (PCA). This enabled the determination of 13 phenolic compounds [133].

Although the latter techniques are useful for the rapid analysis of phenolic compounds in food samples, one drawback is that isomeric compounds cannot be distinguished. Therefore, a separation technique, such as LC, GC and CE coupled to MS, combined with an appropriate extraction method, is the best option to carry out comprehensive characterisation studies of phenolic compounds in complex matrices such as food, nutraceuticals, medicinal plants, etc. The elucidation work depends on the ionisation source, mass analyser, and analytical conditions applied [91]. Besides the latter ionisation sources/interfaces, ESI is generally applied to MS hyphenation with separative techniques such as LC and CE [91,134], while electron impact (EI) ion source (at 70 eV) is the choice for GC-MS. The former is simple, operates at atmospheric pressure and at a moderate temperature [135]. EI is extensively used in GC-Q-MS analysis, and it leads to the fragmentation of molecules. Although it avoids the determination of molecular masses in some cases [136], there are solid standard mass spectral data libraries to characterise compounds on the basis of their fragmentation pattern. Nonetheless, a recent study has shown that GC-TOF-MS with EI enabled not only the detection of fragment ions but also molecular ions (M^+^) found in the spectra. It enabled the measurement of the molecular formula of the compounds with errors up to 10 ppm, which is useful to determine novel compounds or those that are not yet included in databases [137]. Moreover, instead of using both techniques independently, Olmo-García and co-workers proposed the use of both analytical techniques, LC-ESI-MS (using IT-MS and QTOF-MS) and GC-EI-MS when different chemical classes are to be analysed. For example, in olive oil samples, more than forty compounds were identified. Particularly, phenolic compounds, triterpenic compounds, tocopherols and some free fatty acids were identified by LC-MS, while sterols and hydrocarbons were characterised by GC-MS [138]. One of the drawbacks of GC is the necessity of an additional step of derivatisation of the compounds of interest with toxic substances. However, pyrolysis-GC-MS enables the direct analysis of solid samples. Its application in the analysis of phenolic compounds is more marginal because it provides the basic structure of the bioactive compounds through their pyrolysis products produced by thermal degradation. After pyrolysis, these products are separated by GC, ionised by EI and then detected by MS. These compounds can be identified using libraries as for usual GC-MS analysis [139]. This is especially interesting when working with high-molecular-weight compounds, e.g., lignin fragments [139,140], procyanidins [141], etc., since pyrolysis will produce more available fragments for analysis [142].

**Figure 6:**
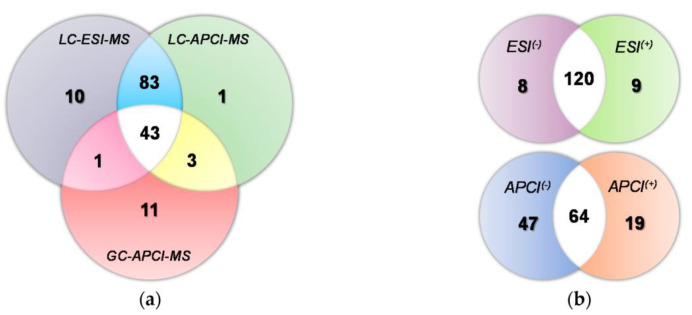
Venn diagrams indicating the total numbers of characterised compounds and those that overlap by (**a**) LC-ESI-MS, LC-APCI-MS and GC-APCI-MS and (**b**) MS polarity, i.e., positive (+) vs. negative (−) ionisation mode by LC-ESI-MS and LC-APCI-MS [143].

Another possible ionisation source is atmospheric pressure chemical ionisation (APCI). It can offer complementary information to that of ESI and EI in LC- and GC-MS analysis. For example, the latter authors applied LC-QTOF-MS coupled to ESI, APCI and GC-APCI-QTOF-MS (after derivatisation) to profile olive extracts from plant materials and olive oil allowing the identification of around 150 compounds [143] (Figure 6). These authors also found that LC-ESI-MS was a very efficient tool for analysing phenolic acids, secoiridoids, flavonoids and lignans, while LC-APCI-MS in the negative mode was appropriate for triterpenic acids and in the positive ionisation mode for sterols and tocopherols. Alternatively, matrix-assisted laser ionisation (MALDI)-TOF-MS can be applied for the characterisation proanthocyanidins [144,145], which mainly comprise flavanols units, as shown by Ricci et al. who analysed food-grade extracts and seeds [144].

**Figure 7:**
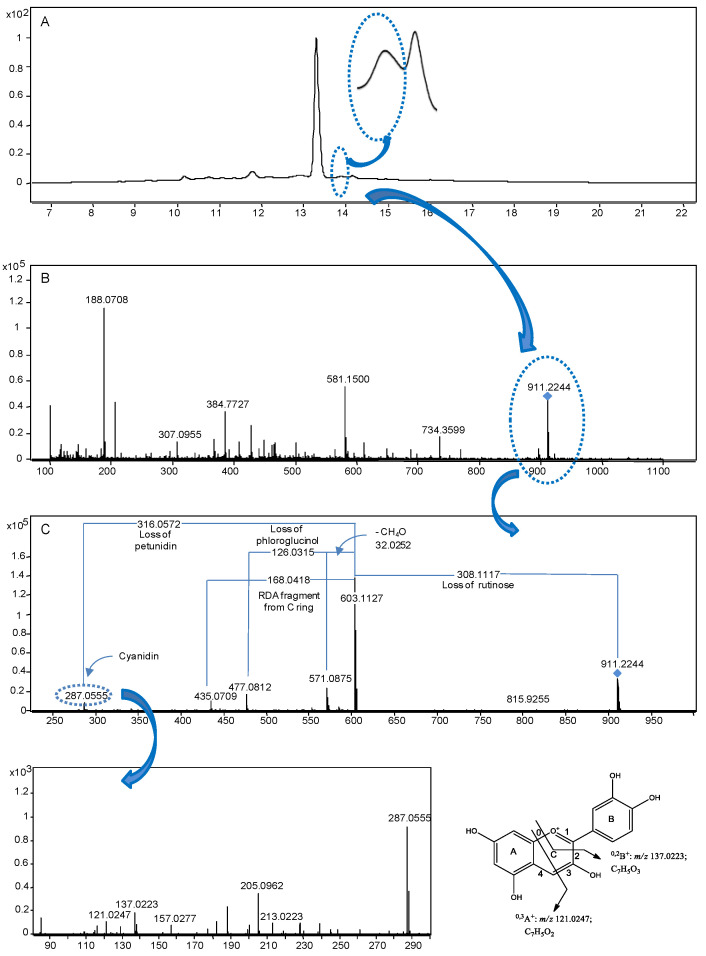
Representation of the strategy followed to characterise the novel dimer of petunidin–cyanidin rutinoside in figs ‘Soltani’ by RP-LC-DAD-QTOF-MS and -MS/MS, which includes (**A**) UV measurement at 520 nm, (**B**) MS spectra evaluation in this region, where the *m*/*z* value of the novel compound is highlighted and (**C**) its main MS/MS fragments, showing neutral losses and the product ions from the fragmentation of the ring C. “Reprinted from *Foods & Function*, 6, Ammar et al., Assessment of the distribution of phenolic compounds and contribution to the antioxidant activity in Tunisian fig leaves, fruits, skins and pulps using mass spectrometry-based analysis, 363, 2015” [102].

LC, GC, and CE coupled to MS provides higher selectivity than, e.g., spectrophotometric detection, although precision is generally inferior [91]. In this sense, by extracting the ion chromatogram (EIC), overlapping and complex peaks can show the compounds present (as an example, see [104]). In most applications, LC-ESI-MS with negative polarity is applied to characterise phenolic compounds. There are numerous examples of its application in food [100,102,103,104,129,146], including novel foods [147], agri-food by-products [148,149], and medicinal plants [105,106,150], as commented before. However, some phenolic compounds, such as furanocoumarins and anthocyanins, are better detected using the positive ionisation mode [102]. These authors tentatively identified a total of 116 compounds, including a novel dimer of petunidin–cyanidin rutinoside, based on the results obtained by RP-LC-DAD-QTOF-MS and -MS/MS, using ESI as the ionisation source in the negative and positive ionisation modes. The characterisation was based on: the retention time, UV–Vis and spectrometric data, including molecular formula, *m*/*z* value, mass error, the isotopic distribution, and MS/MS fragments, which as a puzzle of the molecule, increase the confidence of the tentative identification (as an example, see Figure 7). 

This is of particular importance for novel compounds when commercial standards or information within databases such as Metlin or MassBank are not available. In this sense, the in-depth study of the neutral losses by MS/MS and MS^n^ experiments can give clues about functional groups and moieties: COOH (CO_2_, 44 Da), O (16 Da), OH (H_2_O, 18 Da), methyl (CH_2_/CH_3_, 14/15 Da) and ethyl groups (C_2_H_4_), etc.; *O*-linked sugars, e.g., glucosyl (162 Da), fructosyl (132 Da), etc.; *C*-linked sugars (180 Da, 120 Da, 90 Da, etc.); organic acids, e.g., acetyl (42 Da), malonyl (86 Da), glucuronyl (176 Da), etc.; phenolic moieties, e.g., caffeoyl (162 Da), epi/catechin—H_2_ (288 Da) for B type procyanidins (288 Da), epi/catechin—2H_2_ (286 Da) for A type procyanidins, etc.

Another example of the application of LC-MS in food analysis is through providing information about hydrolytic and oxidative degradation products of phenolic compounds. For that, authors applied RP-LC-ESI-IT-MS and -MS/MS [96,151] and RP-LC-TOF-MS [152] to provide information from the fragmentation pattern and through the measurement of the molecular formula, respectively.

### 3.5. Nuclear Magnetic Resonance Spectroscopy

NMR spectroscopy is commonly used to elucidate the chemical structure of isolated phenolic compounds. Several two-dimensional NMR (2D NMR) experiments are available, including homonuclear experiments as ^1^H–^1^H COSY (correlation spectroscopy) and NOESY (nuclear Overhauser effect spectroscopy), as well as heteronuclear experiments such as HMQC (heteronuclear multiple quantum coherence), ^1^H–^13^C HSQC (heteronuclear single quantum coherence), HMBC (heteronuclear multiple bond coherence), etc. [153]. For example, using NMR-based identification consists of ^1^H-NMR, ^13^C-NMR, COSY, z-filtered TOCSY (total correlated spectroscopy), ROESY (rotating frame Overhauser effect spectroscopy), HMBC and HSQC experiments, a novel *N*-feruloyl tyramine dimer was characterised in a purified fraction from goji berries [154]. Along with IR, another application of NMR is to elucidate how phenolic compounds interact with food matrices, especially, when functionality is investigated [155].

**Figure 8:**
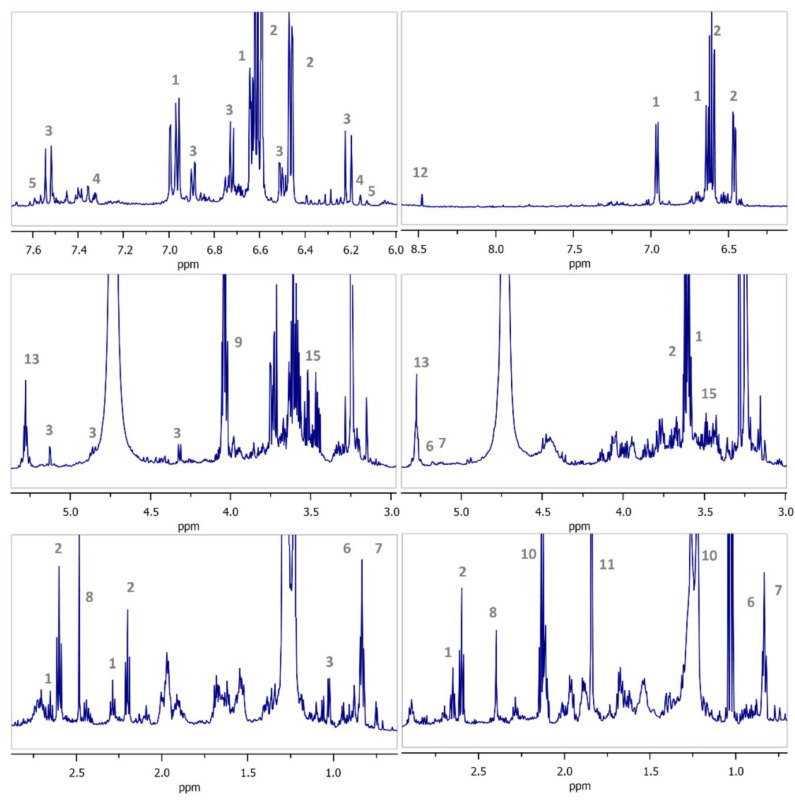
1D ^1^H-NMR spectra from table olives (cultivars ‘Kalamon’ and ‘Chalkidikis’) with different processing methods and geographical origins. (1) Tyrosol; (2) hydroxytyrosοl; (3) verbascoside; (4) luteolin; (5) quercetin; (6) maslinic acid; (7) oleanolic acid; (8) succinic acid; (9) lactic acid; (10) propionic acid; (11) acetic acid; (12) formic acid; (13) triacylglycerol; (14) linoleic acid; and (15) glycerol [158].

NMR also provides chemical profiling of complex mixtures such as food samples, with ^1^H- and ^13^C-NMR commonly applied [156]. For example, using ^1^H-NMR the identification of the *Mentha* genus was confirmed by the presence of rosmarinic acid. It was related to the presence of doublet proton signals with coupling constants at δ 7.49 (d, 15.9 Hz) and δ 6.29 (d, 15.9 Hz) in the aromatic region (δ 8.5–6.0 ppm), which is related to its chemical structure [157]. Additionally, using 1D ^1^H-NMR, phenolic signals from two table olive types, which resonate downfield, researchers showed high variation between their respective spectra (Figure 8) [158]. Moreover, these authors identified marker compounds using chemometrics and statistical total correlation spectroscopy (STOCSY).

As a quantification tool, it seems to show low sensitivity but further chemical treatment, separation devices or appropriate standards are not required [159]. High-resolution multinuclear (^1^H, ^13^C, ^31^P) NMR spectroscopy, 1D-DPFGSE and ^13^C{^1^H} NMR have been applied for the quantification of phenolic compounds, e.g., secoiridoid derivatives in olive oil [159,160].

NMR cannot be considered a routine analytical instrument but coupled to LC-SPE enables the preparation of enriched samples before structural analysis by NMR [161]. This combined with MS or both techniques independently (but complementary used) can increase the number of identified phenolic compounds as shown in a recent study in *Annona cherimola* L. leaves [162]. One interesting trend is its coupling with bioassays, which enables screening of active molecules or markers and elucidating their structure [153]. Additionally, chemometrics can be of help for this purpose. For example, UPLC-DAD-MS-SPE/NMR was applied based on PLS-discriminant analysis (DA) of LC-MS information to elucidate active compounds with lowering cholesterol activity in extracts of crab apples, e.g., hyperoside, myricetin, naringenin, quercetin, kaempferol, among others [161].

### 3.6. Other Technologies

Other technologies are already being applied to elucidate and determine phenolic compounds in foods, nutraceuticals, and plants such as Raman spectroscopy (RS) and ion mobility spectrometry (IMS). While the former is used to detect vibrational, rotational, and other states in a molecular system [163], the latter entails the separation of ions in an inert-buffered gas in the presence of an electric field [164].

**Figure 9:**
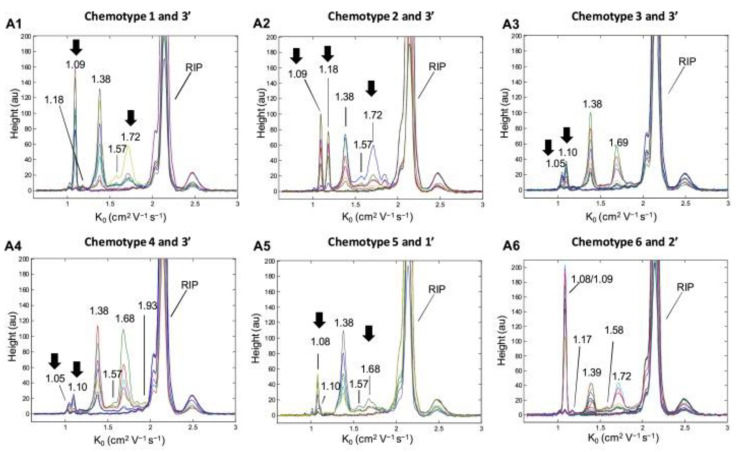
Spectra of different chemotypes of *Cannabis sativa* L. obtained by thermal desorption-ion mobility spectrometry in the positive ionisation mode. The arrows show characteristic signals of the chemotypes. “Reprinted from *Sensors and Actuators B: Chemical*, 273, Contreras et al., Thermal desorption–ion mobility spectrometry: A rapid sensor for the detection of cannabinoids and discrimination of *Cannabis sativa* L. chemotypes, 1413–1424, Copyright (2018), with permission from Elsevier” [166].

As in the other spectroscopy techniques, when RS and IMS are applied alone (without a previous separation technique), chemometrics are required to reveal and analyse the information provided by the RS and IMS spectra. For example, RS together with PLS served to quantify p-hydroxybenzoic acid in honey samples, with better or similar performance than the combination IR and PLS [165]. Concerning IMS, a recent study on cannabinoids, the phenolic components of hemp, enabled their direct analysis in plant solid samples using thermal desorption-IMS. For this, IMS spectra were pre-processed and then, using PCA-linear discriminant analysis, related to their chemotype based on GC-MS [166]. The reduced mobilities values (K_0_), which are a characteristic parameter, measured by this technique could be related to specific cannabinoids after comparison with standards, e.g., K_0_ values at 1.09 cm^2^ V^−1^ s^−1^ (cannabidiol/cannabidiolic acid), 1.18 cm^2^ V^−1^ s^−1^ (cannabidivarin), 1.08 cm^2^ V^−1^ s^−1^ (Δ^9^-tetrahydrocannabinol/Δ^9^-tetrahydrocannabinolic acid), 1.16 cm^2^ V^−1^ s^−1^ (Δ^9^-tetrahydrocannabivarin) and 1.05/1.10 cm^2^ V^−1^ s^−1^ (cannabigerolic acid and/or cannabigerol) (Figure 9).

A promising trend is the coupling of LC, IMS, and MS to give additional and more valuable information, specially, concerning isomeric and isobaric compounds. It also enables the determination of characteristic parameters of compounds, K_0_ values, as mentioned before, and standardised collisional cross-section (CCS) values, which provides an indication of an ion’s size and shape [167,168]. Applied to food samples, hydrophilic interaction chromatography × RP-LC × IMS–MS enables the separation of trimeric procyanidin isomers (*m*/*z* 865) from grape seed that was previously not possible in any other way [167].

## 4. Conclusions

According to the literature consulted, the novel and emerging extraction methods and solvents promote the recovery of phenolic compounds and increase their bio-accessibility. However, studies are needed in relation to the physical and chemical transformations that phenolic compounds suffer during the extraction processing methods. On the other hand, the high structural diversity of these molecules must be considered when these extraction methods are used, and the combination of different techniques may be required for obtaining different families of phenolic compounds that exist. For this purpose, there are a plenty of analytical techniques that can be applied for solid and liquid samples. Among them, mass spectrometry stands out since its versatility to be coupled with chromatography and other detectors such as UV-Vis, FLD, IMS and with different ionisation methods are unrivalled. Nonetheless, more tools and more information will be obtained about this family of valuable compounds in food samples.

## Figures and Tables

**Table 1:** Comparison of different extraction techniques of phenolic compounds. Advantages and drawbacks.

Extraction	Advantages	Disadvantages	Main Applications	References
LLE	Easy to use, efficient and wide-ranging applicability	Poor selectivity, low yields, formation of emulsions and high quantities of organic solvents	To process temperature-sensitive compounds and azeotropic mixtures	[15,16]
SPE	Fast, reproducible, and emulsion-free procedure. Small extract volumes can be used	Time-consuming and high solvent usage	Clean-up method of crude plant extracts	[17]
UAE	Efficient, rapid, selective, and energy-saving technique. Capable of being up-scaled in volume at industrial level	Ultrasound may cause lipid oxidation and formation of free radicals	Useful for thermolabile compounds as it does not require high temperatures	[18,19]
SFE	Rapid and selective, products free of residual solvents	Investment, high pressures, energy costs	Thermolabile compound extraction	[20]
PLE	Faster than conventional extraction techniques, low-solvent consumption	Low selectivity, high temperatures and costly equipment	Extraction of antioxidant phenolic compounds	[21]
MAE	Simple and rapid technique, low-solvent, and energy consumption	Proper selection of power to avoid high temperatures	Suitable for thermolabile phenolic compounds	[22]

Abbreviations: LLE, liquid–liquid extraction; SPE, solid-phase extraction; UAE, ultrasound-assisted extraction; SFE, supercritical fluid extraction; PLE, pressurised liquid extraction and MAE, microwave-assisted extraction.

**Table 2:** Main techniques and extraction systems commonly used for obtaining phenolic compounds from plant materials.

Plant Material	Extraction Technique	Optimised Conditions	Phenolic Compounds	Reference
Rice grains	PLE	Extraction solvent (60% ethyl acetate in methanol), temperature (190 °C), pressure (200 atm) and static time (10 min)	Protocatechuic acid, vanillic acid, vanillin, protocatechuic aldehyde, *p*-hydroxybenzoic acid, *p*-hydroxybenzaldehyde, ferulic acid, sinapic acid, guaiacol, *p*-coumaric acid, caffeic acid, chlorogenic acid, 5-hydroxymethyl-2-furaldehyde, catechin, 5-methylfurfural, ellagic acid and iso-vanillic acid	[23]
*Eucalyptus robusta* leaf	MAE	Water, power (600 W) for 3 min, and 2% (*w*/*v*) solid loading	Phenolic, flavonoid and pro-anthocyanidin compounds	[24]
*Picea* *abies* bark	UAE	53% (*v*/*v*) Methanol, 63 °C, and 38 mL:1 g (dry) for solid loading	Vanillic acid, syringic acid, p-coumaric acid, ferulic acid, sinapinic acid	[25]
Guaraná (*Paullinia cupana*) seeds	SFE	40% Ethanol:methanol for 40 min, 40 °C, and 100 bar	caffeine, catechin, and epicatechin	[26]
Fig fruits	LLE	18% Ethanol (*w*/*w*), 25% K_2_HPO_4_ (*w*/*w*), 10–30 °C, and 3% (*w*/*w*) for solid loading	More than 75% of phenolic compounds were recovered (gallic acid, chlorogenic acid, syringic acid, (+)-catechin, (−)-epicatechin and rutin)	[27]
White grapes	SPE	C_18_ cartridges previously conditioned with 30 mL of methanol and 70 mL of aqueous HCl (pH 2). Phenolic fraction eluted with ethanol	Caftaric acid, coutaric acid, fertaric acid, quercetin and kaempferol and their glycosides (3-*O*-glucoside and 3-*O*-rutinoside)	[28]

Abbreviations: LLE, liquid–liquid extraction; SPE, solid-phase extraction; UAE, ultrasound-assisted extraction; SFE, supercritical fluid extraction; PLE, pressurised liquid extraction and MAE, microwave-assisted extraction.

## Data Availability

Not applicable.
